# Oxidative stress promotes d-GalN/LPS-induced acute hepatotoxicity by increasing glycogen synthase kinase 3β activity

**DOI:** 10.1007/s00011-014-0720-x

**Published:** 2014-02-16

**Authors:** Linlin Wei, Feng Ren, Xiangying Zhang, Tao Wen, Hongbo Shi, Sujun Zheng, Jing Zhang, Yu Chen, Yuanping Han, Zhongping Duan

**Affiliations:** 1 Beijing Artificial Liver Treatments & Training Center, Beijing YouAn Hospital, Capital Medical University, Beijing, China; 2 Beijing Institute of Hepatology, Beijing YouAn Hospital, Capital Medical University, Beijing, China; 3 The College of Life Sciences, Sichuan University, Chengdu, China

**Keywords:** GSK3β, SB216763, Acute liver failure, Oxidative stress, *N*-acetylcysteine

## Abstract

**Objective:**

Our previous studies have demonstrated that glycogen synthase kinase 3β (GSK3β) activity is increased in the progression of acute liver failure (ALF), which aggravates liver injury, while its regulatory mechanism remains elusive. This study is designated to address whether oxidative stress activates GSK3β to promote ALF.

**Methods:**

In a murine model induced by d-galactosamine (d-GalN) (700 mg/kg) and LPS (10 μg/kg), *N*-acetylcysteine (300 mg/kg) or SB216763 (25 mg/kg) was used to inhibit oxidative stress or GSK3β activity, respectively. Serum alanine aminotransferase and aspartate aminotransferase levels were assessed. The parameters of oxidative stress were evaluated in liver tissue. Whether GSK3β inhibition protects hepatocytes from oxidative stress-induced cell apoptosis was investigated in vitro. Moreover, the activity of GSK3β was measured in the liver of chronic hepatitis B (CHB) patients and ALF patients.

**Results:**

In vivo, *N*-acetylcysteine ameliorated the d-GalN/LPS-induced hepatotoxicity and reduced GSK3β activity; GSK3β inhibition increased hepatic superoxide dismutase activity and the glutathione content, decreased malondialdehyde production in the liver tissues; while GSK3β inhibition suppressed the JNK activation in the liver and decreased cytochrome *c* release from mitochondria. In vitro, GSK3β inhibition lessened hepatocytes apoptosis induced by H_2_O_2_ or Antimycin A, as demonstrated by decreased LDH activity, and reduced cleavage of caspase-3 expression. Furthermore, GSK3β activity in the CHB patients was increased in the phase of ALF.

**Conclusions:**

Results indicate that GSK3β activation contributes to liver injury by participating in oxidative stress response in ALF and is, therefore, a potential therapeutic target for ALF.

## Introduction

Acute liver failure (ALF, sometimes referred to as fulminant hepatic failure) is a severe liver disease characterized by encephalopathy and coagulopathy in patients with previously normal liver function [1–3]. There is no effective therapy for ALF other than liver transplantation. In China, ALF is a serious consequence of the acute exacerbation of hepatitis B virus (HBV) infection. To some extent, the ALF has been created by an animal model induced by the co-injection of d-galactosamine (d-GalN) and lipopolysaccharides (LPS), which has been widely used to examine the mechanisms underlying ALF [4, 5].

GSK3 is a ubiquitously expressed serine/threonine kinase that is initially found to regulate glycogen synthesisThere are two highly homologous isoforms, GSK3α and GSK3β. Originally identified in mammals as a cytoplasmic modulator of glycogen metabolism, GSK3β is now recognized as a central regulator of cellular events, including cell fate determination, microtubule function, cell cycle regulation, apoptosis, and inflammatory responses [6–11]. Our previous study showed that GSK3β is activated in the progression of ALF induced by d-GalN/LPS, and moreover, GSK3β inhibition can ameliorate hepatotoxicity in ALF mice [12], while its regulatory mechanism is very poorly understood.

Oxidative stress is a condition in which the cellular levels of reactive oxygen species (ROS) exceed the neutralizing capacity of antioxidants. Injurious oxidant stress, therefore, may result from either the excessive production of ROS, or a diminution in antioxidant levels, and a combination of these two effects [13, 14]. Oxidative stress is common in various types of liver injury, and plays a critical role in the mechanism of ALF [15]. Recent studies have revealed that oxidative stress may also regulate cell death by altering GSK3β activity. For instance, a critical role of GSK3β is found in mediating the oxidative stress-induced neuronal cell death [16–19]. The stress-induced GSK3β regulates the redox stress response by phosphorylating glucose-6-phosphate dehydrogenase, as demonstrated in arabidopsis [20].

Given the notion that oxidative stress can regulate the GSK3β pathway to promote the cell apoptosis, we hypothesized that oxidative stress-GSK3β may be play a important role in the mechanism of d-GalN/LPS-induced ALF. To test this hypothesis, we performed experiments to evaluate the oxidative stress during the progression of ALF, to investigate GSK3β inhibition in regulating oxidative stress through both the in vivo and in vitro models. Moreover, to confirm their clinical relevance, we further evaluated the activity of GSK3β in ALF induced by HBV infection.

## Methods

### Animals and treatment

Male wild-type (WT, C57BL/6) mice (8–12 weeks of age) were purchased from Capital Medical University (Beijing, China) and housed in the Capital Medical University animal facility under specific pathogen-free conditions, and received humane care according to Capital Medical University Animal Care Committee guidelines. To induce ALF, the mice were injected intraperitoneally with d-GalN (700 mg/kg, Sigma, St Luis, MO) and LPS (10 μg/kg, Invivogen, San Diego, CA). Mice were treated with a single dose of freshly prepared *N*-acetylcysteine (NAC, at 300 mg/kg, by i.p. injection, Sigma, St Luis, MO), a ROS scavenger, immediately after d-GalN/LPS treatment. In some experiments, SB216763 (25 mg/kg, Sigma, St Luis, MO) dissolved in dimethyl sulfoxide (DMSO), a specific inhibitor for GSK3β, was suspended in phosphate-buffered saline (PBS) and administered intraperitoneally 2 h prior to d-GalN/LPS treatment. Mice were sacrificed at various time-points after d-GalN/LPS treatment; liver and serum samples were collected for future analysis.

### Serum aminotransferase activities

Serum levels of alanine aminotransferase (ALT) and aspartate aminotransferase (AST), as markers of hepatic damage, were measured by using a multiparameteric analyzer (AU 5400, Olympus, Japan), according to an automated procedure.

### Assay of ROS level in liver

To determine the level of ROS in the liver tissue, liver homogenates were made in lysis buffer and analyzed using a chemical fluorescent ROS assay kit (Jiancheng Bio, Nangjing) according to the manufacturer’s instruction, which is based on the oxidation of 2′7′-dichlorodihydrofluorescein diacetate to 2′7′-dichloro-fluorescein.

### Assay of MDA,GSH and SOD in liver

A part of liver tissue was prepared for homogenization with a buffer containing 0.15 M KCl to obtain 1:10 (w/v) homogenates. The homogenates were then centrifuged at 12,000×*g* (4 °C) for 20 min to collect the supernatants. The concentrations of malondialdehyde (MDA), and glutathione (GSH), as well as superoxide dismutase (SOD) activities were measured, as described previously [21].

### Cell cultures

Human hepatocyte line HL-7702 (Xiangfu Biological Company, Shanghai) was plated in 48-well or 6-well plates at an appropriate density. After overnight culturing, hydrogen peroxide (H_2_O_2_, 1 mmol/L, Habo Company, Shanghai) or antimycin A (2 μg/ml, Sigma, St Luis, MO) was added into the culture wells. To study the effects of GSK3β inhibition on hepatocyte apoptosis induced by oxidative stress, SB216763 (10 mΜ) was added 2 h prior to the H_2_O_2_ or antimycin A treatment. Cell apoptosis was evaluated at 12 h by caspase western blots and lactate dehydrogenase (LDH) assay (Biochain Institute, Hayward, CA) of culture supernatants, according to the manufacturer's instructions.

### Western blot analysis

Protein was extracted from liver tissue with RIPA buffer with phosphatase inhibitors and protease inhibitors. Proteins in sodium dodecyl sulfate (SDS)-loading buffer were subjected to SDS-12 % polyacrylamide gel electrophoresis (PAGE) and transferred to polyvinylidene difluoride (PVDF) membrane. Monoclonal rabbit antibodies against p-GSK3β, total GSK3β, p-JNK, total JNK, Cyt *c*, caspase-3, cleaved caspase 3 and β-actin (Cell Signaling Technology, Santa Cruz, CA) were used at 4 °C overnight. Then the membrane was treated with horseradish peroxidase-conjugated goat anti-rabbit secondary antibody. The relative quantities of proteins were determined by a densitometer and expressed as absorbance units (AU).

### Immunofluorescence staining

Paraffin sections were treated with xylene for three times with 10 min each time. The sections were hydrated through a graded alcohol series and then rinsed three times with distilled water. The slides were incubated for 20 min in 10 % goat serum in PBS and then p-GSK3β rabbit monoclonal antibody (Cell Signaling Technology, Santa Cruz, CA) overnight at 4 °C. The slides were incubated with Alexa Fluor^®^ 568 goat anti-rabbit IgG (1:200, Invitrogen, Grand Island, NY) for 45 min. After three washings with PBS, the nuclei were stained with 4′,6-diamidino-2-phenylindole (DAPI, 1 μg/ml, Shizebio, Shanghai) for 10 min. The images were examined on a Nikon Eclipse E800 fluorescent microscope.

### Determination of hepatic GSK3β activity

To determine the activity of GSK3β in the liver tissue, liver homogenates were made in lysis buffer and analyzed using a colorimetric GSK3β assay kit (Jianglaibio Co, Shanghai) according to the manufacturer’s instructions.

### Human liver samples

This study meets the ethical guidelines of the 1975 Declaration of Helsinki and the study protocol was permitted by the Medical Ethics Committee of Beijing YouAn Hospital. Informed consent was obtained from all patients. Human liver tissue was obtained from the normal subjects, chronic hepatitis B (CHB) patients and HBV-induced ALF patients. All patients were hospitalized during 2009–2011 at Beijing YouAn Hospital. Clinical characteristics and details of clinical data of the patients in analysis are shown in Table 1.

**Table 1:** General clinical characteristics of the different study groups

	Normal subjects (*n* = 8)	Chronic hepatitis B patients (*n* = 12)	Acute liver failure patients (*n* = 12)	*P* value
Age (years)	39.4 ± 3.6	32.4 ± 4.1	41.6 ± 5.3	0.11
Gender (male/female)	6/2	7/5	8/4	0.437
Alanine aminotransferase (U/L)	35.1 ± 6.1	93.3 ± 18.3	210.4 ± 76.3	0.026
Aspartate aminotransferase (U/L)	30.6 ± 3.9	62.6 ± 15.4	305.8 ± 44.6	0.038
Serum bilirubin (μmol/l)	8.8 ± 2.9	20.9 ± 6.3	196.1 ± 50.6	0.01
Prothrombin time (s)	9 ± 2.4	15 ± 5.2	34.4 ± 7.2	0.022
Albumin (g/L)	46.2 ± 6.9	32.6 ± 10.8	26.7 ± 5.7	0.039
Creatinin (μmol/L)	73.6 ± 21.5	80.1 ± 29.0	95.4 ± 32.8	0.042
Hepatic encephalopathy score	−	−	1.7 ± 0.3	−
Child-Pugh score	−	6 ± 0.6	13.7 ± 2.4	0.031
Model for end-stage liver disease score	−	−	28.2 ± 4.2	−
HBsAg test	−	Positive	Positive	−

### Statistical analysis

Results are shown as mean ± SD. The data were analyzed with statistics software SPSS 11.5 using a nonparametric analysis of variance test. Differences were considered significant if the *P* value was less than 0.05.

## Results

### Imbalance of oxidation and antioxidant in the d-GalN/LPS-induced ALF

**Fig. 1:**
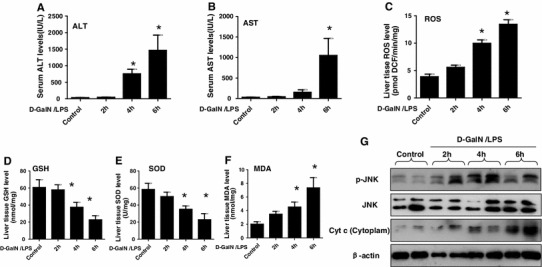
Imbalance of oxidation and antioxidant in the d-GalN/LPS-induced ALF. The mice were injected intraperitoneally with d-GalN (700 mg/kg) and LPS (10 μg/kg) for 2, 4 and 6 h (10 mice/groups), the mice of control group (*n* = 8) were injected with only PBS. **a**, **b** The serum ALT and AST levels were analyzed at different time points after d-GalN/LPS. *p < 0.05 compared with control. **c–f** The parameters of oxidative stress including ROS, GSH, SOD and MDA were measured at different time points after d-GalN/LPS. * *p* < 0.05 compared with control. **g** The p-JNK, total JNK, β-actin levels and Cyt *c* protein levels in cytoplasm released from mitochondria were measured by western blot at different time points after d-GalN/LPS. Representatives of two experiments are shown

To evaluate the role of oxidant stress in the pathogenesis of ALF, we first determined in vivo whether the parameters of oxidative stress were triggered in liver. The serum ALT and AST levels reached 1462.58 ± 587.12 and 1398.65 ± 437.23 IU/L at 6 h after d-GalN/LPS injection, while the serum ALT and AST levels of control group treated with PBS at 6 h were 45.5 ± 11.7 and 31.8 ± 9.2 IU/L, respectively (Fig. 1a, b). With the progression of liver injury, d-GalN/LPS administration increased ROS and MDA concentrations but decreased SOD activities and GSH contents markedly in comparison with the control mice from 4 to 6 h (Fig. 1c–f). The previous study had shown that the activated JNK pathway played an important role in oxidative stress of ALF [12, 22], and leakage of mitochondrial contents, such as cytochrome *c* (Cyt *c*), was considered as a major event in cellular apoptosis [23], so we next determined whether leakage of Cyt *c* from the mitochondria and p-JNK took place during ALF development. Relative to protein expression of total JNK and β-actin, the levels of p-JNK and Cyt *c* (in cytoplasm) were increased significantly from 4 to 6 h after d-GalN/LPS administration (Fig. 1g). These results suggested that oxidant stress was triggered in d-GalN/LPS-induced ALF.

### NAC, an antioxidant, protects mice from liver injury and decreases GSK3β activity

**Fig. 2:**
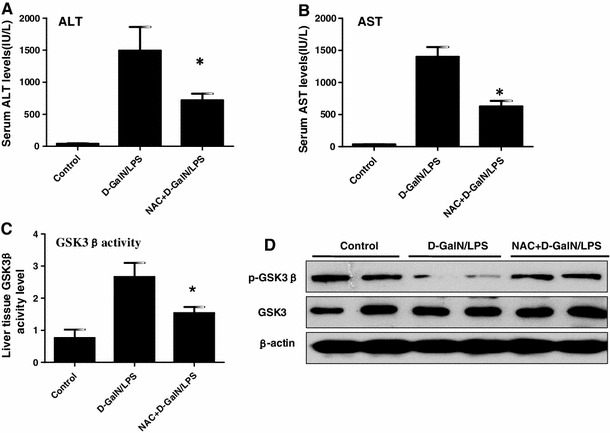
NAC protects mice from liver injury and decreases GSK3β activity. The mice of NAC+ GalN/LPS groups were injected intraperitoneally with NAC (300 mg/kg) immediately after d-GalN/LPS injection (*n* = 10/group); the mice of GalN/LPS groups were injected intraperitoneally with d-GalN/LPS (*n* = 10/group); the mice of control group (*n* = 8) were injected with only PBS. The serum and liver samples were harvested at 6 h after d-GalN/LPS injection. **a**, **b** The serum ALT and AST levels were analyzed at 6 h after d-GalN/LPS. *p < 0.05 compared with the d-GalN/LPS injection group. **c** The activity of GSK3β in the liver tissue was measured in three different groups. *p < 0.05 compared with d-GalN/LPS injection group. **d** The expression of p-GSK3β, total GSK3β and β-actin proteins were measured by western blots. Representative of two experiments are shown

*N*-acetylcysteine (NAC) is a glutathione precursor, which increases glutathione levels in hepatocytes, and in turn, limit the production of ROS, which directly cause hepatocellular injury [24]. We, therefore, wanted to demonstrate whether antioxidant of NAC can regulate GSK3β activity in d-GalN/LPS-induced ALF. Compared to control groups, livers in mice receiving NAC treatment suffered less liver injury, evidenced by distinct lower sALT and sAST levels (Fig. 2a, b). Meantime, the activity of GSK3β was inhibited by NAC in the ALF model (Fig. 2c); moreover, the phosphorylated (serine 9) GSK3β level in the liver tissues was promptly increased in NAC-treated mice which also meant that GSK3β activity was reduced (Fig. 2d). Thus, the NAC exerted protection of the mice from liver injury was associated with anti-oxidative stress, and attenuation of GSK3β activity as well.

### GSK3β inhibition blocks d-GalN/LPS-induced oxidative stress

**Fig. 3:**
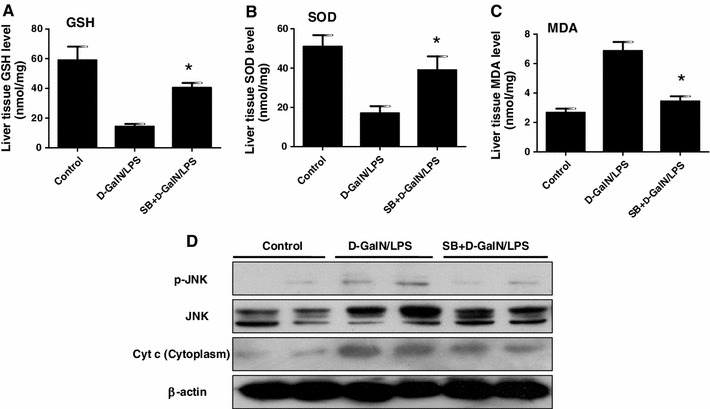
GSK3β inhibition blocks d-GalN/LPS-induced oxidative stress. The mice of the SB+ GalN/LPS groups were injected intraperitoneally with SB216763 (25 mg/kg) prior to d-GalN/LPS treatment (*n* = 10/group); the mice of GalN/LPS groups were injected intraperitoneally with d-GalN/LPS (*n* = 10/group); the mice of control group (*n* = 8) were injected with only PBS. The serum and liver samples were harvested at 6 h after d-GalN/LPS injection. **a**, **b**, **c** The parameters of oxidative stress including GSH, SOD and MDA were measured in different groups. *p < 0.05 compared with the d-GalN/LPS injection group. **d** The p-JNK, total JNK, β-actin levels and Cyt *c* protein levels in cytoplasm released from mitochondria were measured by western blot at different time points after d-GalN/LPS. Representatives of two experiments are shown

Our previous study had shown that GSK3β was activated in the progression of ALF and GSK3β inhibition protected the liver injury induced by d-GalN/LPS [12]. Next, we wanted to explore whether the GSK3β inhibition had effects on the oxidative stress in the ALF model. Indeed, SB216763-pretreatment for 2 h can partially restore levels of GSH and SOD, and suppress the level of MDA (Fig. 3a–c). Moreover, western blot results showed that the expression of p-JNK in liver was decreased by SB216763, while the expression of total JNK was comparable; the level of Cyt *c* protein released from liver mitochondria into cytoplasm was also decreased by SB216763 (Fig. 3d). Therefore, these results demonstrated that GSK3β inhibition was capable of inhibiting oxidative stress in ALF induced by d-GalN/LPS.

### GSK3β activity is triggered by oxidant stress in vitro

**Fig. 4:**
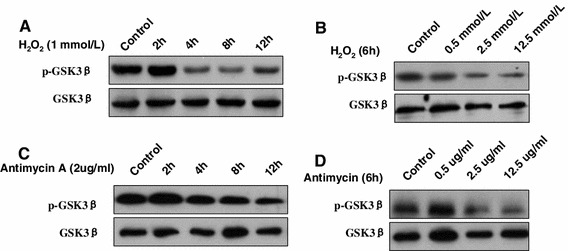
GSK3β activity is triggered by oxidant stress. HL-7,702 cells were incubated with H_2_O_2_ or Antimycin A at different times or different doses. HL-7,702 cells incubated with only PBS as a control. **a**, **b** The lysates of cells stimulated with H_2_O_2_ were analyzed for p-GSK3β (serine 9), total GSK3β and β-actin levels by western blot. **c **, **d** The lysates of cells stimulated with Antimycin A were analyzed for p-GSK3β (serine 9), total GSK3β and β-actin levels by western blot

Here, we further examined whether the oxidant stress induced GSK3β activity through an in vitro model. Western blot results showed that H_2_O_2_-inducing oxidant stress promoted GSK3β activity as indicated by dephosphorylation at serine 9, in a time-dependent and dose-dependent manner (Fig. 4a, b). Previous studies have demonstrated that Antimycin A, an inhibitor of electron transport in mitochondria, had been used as a ROS generator in biological systems [25, 26]. We further examined whether GSK3β activity is increased in response to oxidant stress. As shown in Fig. 4c, d, GSK3β activities were clearly also increased by Antimycin A in a time-dependent and dose-dependent manner. Therefore, we demonstrated that GSK3β was explicitly activated by oxidant stress.

### GSK3β inhibition attenuated oxidant stress-induced hepatocyte apoptosis

**Fig. 5:**
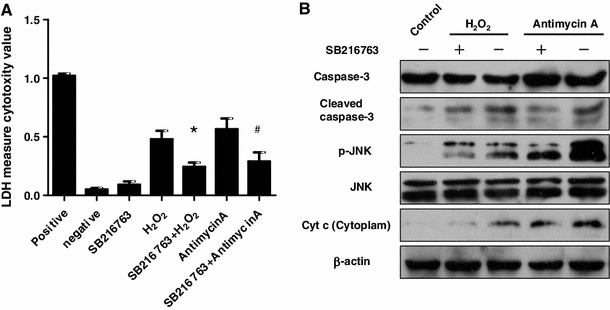
GSK3β inhibition attenuated oxidant stress-induced hepatocyte apoptosis. **a** LDH activities assay in the HL-7,702 cell culture supernatant. Positive control group: the maximum LDH release induced by the addition of 1× lysis solution. Negative control group: the LDH activity released from the untreated normal cells. SB216763 group: the LDH activity released from the solely SB216763-treated for 12 h. H_2_O_2_ group and SB263763+ H_2_O_2_ group: cells were pre-incubated with or without SB216763 (10 mΜ) for 2 h, then incubated H_2_O_2_ (1 mmol/L) for 12 h. Antimycin A group and SB263763+ Antimycin A group: cells were pre-incubated with or without SB216763 (10 mΜ) for 2 h, then incubated with Antimycin A (2 μg/ml) for 12 h. All cells in the groups are in the 48 wells culture plates (3 wells/group). *p < 0.05 compared with H_2_O_2_-treated cells; ^#^ *p* < 0.05 compared with Antimycin A-treated cells. **b** The expression of caspas-3, cleaved caspase-3, p-JNK, total JNK, Cyt *c* and β-actin proteins was measured by western blot. Pre-incubated with or without SB216763 (10 mΜ) for 2 h, then HL-7,702 cells are stimulated with H_2_O_2_ (1 mmol/L) or Antimycin (2 μg/ml) for 12 h. The control group is the untreated normal cells. Cell lysates were subject to western blot with antibodies. Representatives of experiments are shown

As shown above, oxidant stress played an important role in the mechanism of d-GalN/LPS-induced ALF, so we investigated the role of GSK3β in the intrinsic potential of hepatocyte apoptosis triggered by oxidant stress in vitro. As shown, H_2_O_2_ or Antimycin A treatment can substantially increase the release of LDH from the human hepatocyte cells; in contrast, inhibition GSK3β with SB216763 significantly decreased LDH levels of hepatocyte treated by H_2_O_2_ or Antimycin A (Fig. 5a). Western blot analysis also showed that SB216763 inhibited the expression of cleaved caspase-3, as compared with H_2_O_2_ or Antimycin A-treated cells; furthermore, SB216763 also decreased p-JNK in liver and the Cyt *c* protein level in cytoplasm, but did not influence the expression of total JNK and β-actin (Fig. 5b). Therefore, GSK3β inhibition was capable of reducing the hepatocyte apoptosis induced by oxidant stress.

### Increased GSK3β activity in liver of ALF patients

**Fig. 6:**
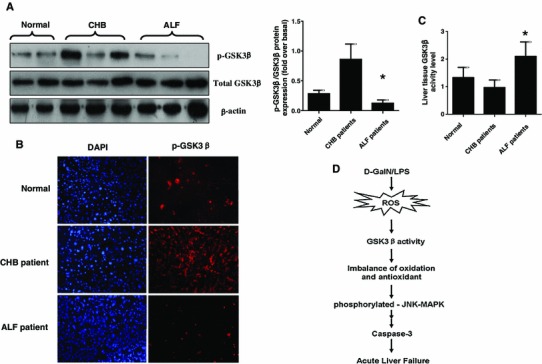
GSK3β activation is decreased in CHB patients and increased in ALF patients. **a** Hepatic proteins expression of phosphorylated (serine 9) GSK3β, total GSK3β and β-actin were measured by western blots in normal, CHB patients and ALF patients. Representatives of experiments are shown. Densitometry analysis of the proteins was performed for each sample (mean ± SD). **b** Immunofluorescence staining for phosphorylated GSK3β (*red*) in the liver of patients. Representatives of experiments are shown. Original magnification ×200. **c** The activity of GSK3β in the liver tissue was measured in three different groups. *p < 0.05 compared with normal group. **d** In the d-GalN/LPS-induced ALF mice model, oxidative stress is triggered which promotes activity of GSK3β and further breaks the balance of oxidation and antioxidant. These events ultimately leads to incremental hepatocyte apoptosis, which induces by oxidative stress in liver and induces the development of ALF (color figure online)

To determine whether GSK3β participates in progression of ALF patients, we utilized liver tissue to measure the changes of GSK3β activity among the normal subjects, CHB patients and ALF patients induced by HBV. Relative to the normal controls, western blot analysis showed that the levels of p-GSK3β (at serine 9) were slightly increased in CHB, but significantly reduced in ALF (Fig. 6a). The similar results were obtained by immunofluorescence staining (Fig. 6b), and measurements of GSK3β activities in liver tissue (Fig. 6c). These results indicated that the constitutively active GSK3β in the liver was restrained in CHB patients, while increased in ALF patients.

## Discussion

The main findings in this study were that oxidative stress played a critical role in the mechanism of ALF, and oxidative stress inhibition by NAC ameliorated the toxin induced liver failure. Importantly, the present study highlighted a novel role of GSK3β in response to oxidant stress in ALF. GSK3β inhibition blocked d-GalN/LPS-induced oxidative stress and was capable of reducing the hepatocyte apoptosis induced by oxidant stress. Therefore, our findings suggest that oxidant stress exacerbates hepatotoxicity in ALF via increasing GSK3β activity (as shown by Fig. 6d).

Oxidative stress is commonly associated with a number of liver diseases, and is thought to play a role in the pathogenesis of ALF [27, 28]. Oxidative stress is a condition in which the cellular levels of ROS exceed the neutralizing capacity of antioxidants [15]. The effects of oxidative stress are balanced by antioxidant activities involving a variety of enzymatic and non-enzymatic mechanisms. Active oxygen scavenging systems include enzymes such as SOD, glutathione peroxidase (GSH-P_X_) and catalase, and non-enzymatic antioxidants including GSH, vitamin C, and vitamin E, etc. [29]. Our results have shown that the activities of SOD and GSH in ALF are significantly lower than the normal control, which are negatively correlated with serum ALT and AST levels. Furthermore, the concentration of MDA, which is used as an important indicator of oxidation, was significantly higher than normal values. With the progression of ALF, the status of oxidation is increased, and conversely, the antioxidants gradually reduced, which results in an imbalance of oxidation over anti-oxidation, and the severe oxidative stress in the occurrence of ALF and further progression of liver injury.

Oxidative stress regulates hepatocyte injury and death by altering signal transduction pathways. Studies have shown that ROS may also activate cellular signal pathways, such as mitogen-activated protein kinase (MAPK), nuclear factor-κB (NF-κB), phosphatidylinositol 3-kinase (PI3K), p53, and β-catenin/Wnt, etc. [15, 30–33]. Our study found that GSK3β is a new fulcrum to regulate oxidative stress in ALF. GSK3β is constitutively active in resting cells to a proper extent. GSK3β activity is depressed at an early stage and subsequently returns to a high level in the ALF, which suggests the activity of GSK3β is precisely mediated in the progression of ALF and plays a complicated role in the pathogenesis of ALF. In vitro, oxidative stress activates GSK3β, and GSK3β inhibition significantly alleviates the hepatocyte apoptosis induced by oxidative stress. So, it can be speculated that GSK3β is one of the regulatory molecules of the oxidative stress signaling pathway. In vivo, our results show that the NAC inhibits oxidative stress to protect liver from d-GalN/LPS-induced acute injury in mice, which is in line with the reduced the activity of GSK3β. These results further prove that GSK3β is one of the key regulatory molecules in the balance of oxidation/antioxidant. In summary, we conclude that the balance of oxidation/antioxidant is tilted in the progression of ALF; the oxidation process is in a dominant position because of changes of GSK3β activity, which result in oxidative stress promoting liver injury in ALF. Thus, inhibition of GSK3β activity can reverse the imbalanced oxidation in liver tissue, and GSK 3β is a fulcrum to regulate oxidative stress in ALF.

Previous study had demonstrated that HBV infection increased phosphor ser-9-GSK3β and decreased the GSK3β activity in the Huh7 cell line, which could deliver a replication competent HBV genome [34]. Our studies further explored the issue and showed that GSK3β activity is slightly suppressed in the CHB patients, while it is up-regulated in the ALF patients with HBV infection. It can be speculated that the replication of HBV is in a dominant position and the balance of oxidation/antioxidant has not yet been broken in the patients with chronic hepatitis. At this period, anti-oxidation is in an advantageous position and the activity of GSK3β is affected by HBV. But for ALF patients, the oxidation is in an increasingly dominant position, and oxidative stress may contribute to the increased activity of GSK3β, therefore, either in the d-GalN/LPS-induced ALF model, or in the HBV-induced ALF patients, GSK3β activity is elevated in all. Based on the findings, we speculate that GSK3β activity can be measured to predict the prognosis of HBV infection for the breakthrough and persistence. The in-depth research in these regards is needed as future work.

We need to further clarify that, in hepatitis B virus (HBV) infection, hepatocyte injury is triggered by immune-mediated apoptosis and continues as a nonspecific necroinflammatory response. Necroinflammation of the liver produces excessive oxidative stress leading to further hepatocyte damage. Although oxidative stress plays important roles in HBV-related liver injury, clinically it is not the only factor of hepatotoxicity in CHB. Decrease of GSK3β activity in the liver tissues of CHB patients may be related to a protective response against oxidative stress or inflammatory pressure caused by immune cytotoxicity, as well as HBV itself. Moreover, GSK3β activity may be also regulated by glucose metabolism including glycogen synthesis or insulin interaction under liver injury. Thus, oxidative stress is not the only way to regulate the activity of GSK3β.

In summary, oxidative stress-activated GSK3β may be one of the mechanisms governing the pathological basis for ALF, and GSK3β inhibition represents a potent strategy to suppress oxidative stress, and ameliorate liver pathology. Further preclinical studies with GSK3β inhibitors are warranted to pave the way for the development of a clinically applicable therapeutic strategy against ALF.

## Abbreviations

ALF: Acute liver failure
HBV: Hepatitis B virus
CHB: Chronic hepatitis B
GSK3β: Glycogen synthase kinase 3β
d-GalN: d-galactosamine
LPS: Lipopolysaccharide
ROS: Reactive oxygen species
DMSO: Dimethyl sulfoxide
PBS: Phosphate-buffered saline
NAC: *N*-acetylcysteine
ALT: Alanine aminotransferase
AST: Aspartate aminotransferase
MDA: Malondialdehyde
GSH: Glutathione
SOD: Superoxide dismutase
H_2_O_2_: Hydrogen peroxide
LDH: Lactate dehydrogenase
Cyt c: Cytochrome *c*
SDS: Sodium dodecyl sulfate
PAGE: Polyacrylamide gel electrophoresis;
DAPI: 4′,6-diamidino-2-phenylindole

## References

[1] Hoofnagle JH, Carithers RLJ, Shapiro C, Ascher N (1995). Acute hepatic failure: summary of a workshop. Hepatology.

[2] Gunning K (2009). Hepatic failure. Anaesth Intensive Care.

[3] Lee WM (2012). Recent developments in acute liver failure. Best Pract Res Clin Gastroenterol.

[4] Mignon A, Rouquet N, Fabre M, Martin S, Pagès JC, Dhainaut JF (1999). LPS challenge in D-galactosamine-sensitized mice accounts for caspase-dependent fulminant hepatitis, not for septic shock. Am J Respir Crit Care Med.

[5] Nakama T, Hirono S, Moriuchi A, Hasuike S, Nagata K, Hori T (2001). Etoposide prevents apoptosis inmouse liver with d-GalN/LPS-induced fulminant hepatic failure resulting inreduction of lethality. Hepatology.

[6] Rayasam GV, Tulasi VK, Sodhi R, Davis JA, Ray A (2009). Glycogen synthase kinase 3: more than a namesake. Br J Pharmacol.

[7] Beurel E, Michalek SM, Jope RS (2010). Innate and adaptive immune responses regulated by glycogen synthase kinase-3 (GSK3). Trends Immunol.

[8] Rehani K, Wang H, Garcia CA, Kinane DF, Martin M (2008). IFN-beta production by TLR4-stimulated innate immune cells is negatively regulated by GSK3-beta. J Immunol.

[9] Martin M, Rehani K, Jope RS, Michalek SM (2005). Toll-like receptor-mediated cytokine production is differentially regulated by glycogen synthase kinase 3. Nat Immunol..

[10] Hoeflich KP, Luo J, Rubie EA, Tsao MS, Jin O, Woodgett JR (2000). Requirement for glycogen synthase kinase-3β in cell survival and NF-kappaB activation. Nature.

[11] Eleonore B, Richard SJ (2006). The paradoxical pro-and anti-apoptotic actions of GSK3 in the intrinsic and extrinsic apoptosis signaling pathways. Prog Neurobiol.

[12] Chen L, Ren F, Zhang H, Wen T, Piao Z, Zhou L (2012). Inhibition of glycogen synthase kinase 3β ameliorates d-GalN/LPS-induced liver injury by reducing endoplasmic reticulum Stress-triggered apoptosis. PLoS One.

[13] Dryden GW, Deaciuc I, Arteel G, McClain CJ (2005). Clinical implications of oxidative stress and antioxidant therapy. Curr Gastroenterol Rep.

[14] Han D, Hanawa N, Saberi B, Kaplowitz N (2006). Mechanisms of liver injury. III. Role of glutathione redox status in liver injury. Am J Physiol Gastrointest Liver Physiol.

[15] Czaja Mark J (2007). Cell signaling in oxidative stress-induced liver injury. Semin Liver Dis.

[16] Lee KY, Koh SH, Noh MY, Park KW, Lee YJ, Kim SH (2007). Glycogen synthase kinase-3β activity plays very important roles in determining the fate of oxidative stress-inflicted neuronal cells. Brain Res.

[17] Koh SH, Kim SH, Kwon H, Park Y, Kim KS, Song CW (2003). Epigallocatechin gallate protects nerve growth factor differentiated PC12 cells fromoxidative-radical-stress-induced apoptosis through its effect on phosphoinositide 3-kinase/Akt and glycogen synthase kinase-3. Mol Brain Res.

[18] Koh SH, Kim SH, Kwon H, Kim JG, Kim JH, Yang KH (2004). Phosphatidylinositol-3 kinase/Akt and GSK-3 mediated cytoprotective effect of epigallocatechin gallate on oxidative stress-injured neuronal-differentiated N18D3 cells. Neurotoxicology.

[19] Schäfer M, Goodenough S, Moosmann B, Behl C (2004). Inhibition of glycogen synthase kinase 3β is involved in the resistance to oxidative stress in neuronal HT22 cells. Brain Res.

[20] Dal Santo S, Stampfl H, Krasensky J, Kempa S, Gibon Y, Petutschnig E (2012). Stress-induced GSK3 regulates the redox stress response by phosphorylating glucose-6-phosphate dehydrogenase in arabidopsis. Plant Cell..

[21] Wu ZM, Wen T, Tan YF, Liu Y, Ren F, Wu H (2007). Effects of salvianolic acid A on oxidative stress and liver injury induced by carbon tetrachloride in rats. Basic Clin Pharmacol Toxicol.

[22] Jiyoung H, Margitta L, Anwar F, Hartmut J (2009). Oxidant stress-induced liver injury in vivo: role of apoptosis, oncotic necrosis, and c-Jun NH -terminal kinase activation. Am J Physiol Gastrointest Liver Physiol.

[23] Ying J, Miaoyun Z, Wei A (2011). Increased hepatic apoptosis in high-fat diet-induced NASH in rats may be associated with downregulation of hepatic stimulator substance. J Mol Med.

[24] Pastor A, Collado PS, Almar M, González-Gallego J (1997). Antioxidant enzyme status in biliary obstructed rats: effects of *N*-acetylcysteine. J Hepatol.

[25] Park WH, Han YW, Kim SH, Kim SZ (2007). An ROS generator, antimycin A, inhibits the growth of HeLa cells via apoptosis. J Cell Biochem.

[26] Han YH, Kim SH, Kim SZ, Park WH (2008). Antimycin A as a mitochondria damage agent induces an S phase arrest of the cell cycle in HeLa cells. Life Sci.

[27] Martindale JL, Holbrook NJ (2002). Cellular response to oxidative stress: signaling for suicide and survival. J Cell Physiol.

[28] Ha HL, Shin HJ, Feitelson MA, Yu DY (2010). Oxidative stress and antioxidants in hepatic pathogenesis. World J Gastroenterol.

[29] Zhu R, Wang Y, Zhang L, Guo Q (2012). Oxidative stress and liver disease. Hepatol Res.

[30] Czaja MJ, Liu H, Wang Y (2003). Oxidant-induced hepatocyte injury from menadione is regulated by ERK and AP-1 signaling. Hepatology.

[31] Rosseland CM, Wierød L, Oksvold MP, Werner H, Ostvold AC, Thoresen GH (2005). Cytoplasmic retention of peroxide-activated ERK provides survival in primary cultures of rat hepatocytes. Hepatology.

[32] Gunawan BK, Liu ZX, Han D, Hanawa N, Gaarde WA, Kaplowitz N (2006). C-Jun N-terminal kinase plays a major role in murine acetaminophen hepatotoxicity. Gastroenterology.

[33] Jones BE, Lo CR, Liu H, Pradhan Z, Garcia L, Srinivasan A (2000). Role of caspases and NF-kB signaling in hydrogen peroxide- and superoxide-induced hepatocyte apoptosis. Am J Physiol Gastrointest Liver Physiol.

[34] Chin R, Earnest-Silveira L, Koeberlein B, Franz S, Zentgraf H, Dong X (2007). Modulation of MAPK pathways and cell cycle by replicating hepatitis B virus: factors contributing to hepatocarcinogenesis. J Hepatol.

